# Online Shopping Addiction in Association with Depression, Anxiety, and Stress: Exploring Independent and Relative Contributions in a Cross-Sectional Study

**DOI:** 10.3390/bs16081441

**Published:** 2026-08-20

**Authors:** Mehmet Emin Arayici, Gizem Gedik, Dogan Basaran, Hatice Simsek, Sema Gultekin Arayici

**Affiliations:** 1 Department of Biostatistics and Medical Informatics, Faculty of Medicine, Dokuz Eylül University, 15 July Medicine Campus, Inciralti-Balcova, 35340 Izmir, Türkiye; 2 Department of Public Health, Faculty of Medicine, Dokuz Eylül University, 15 July Medicine Campus, Inciralti-Balcova, 35340 Izmir, Türkiye; 3 Department of Clinical Psychology, Institute of Social Sciences, Ege University, 35100 Izmir, Türkiye; 4 Independent Researcher, 35100 Izmir, Türkiye

**Keywords:** online shopping addiction, compulsive buying, depression, anxiety, stress, DASS-21, behavioral addiction

## Abstract

Psychological distress is associated with problematic online shopping, but the relative and independent associations of depression, anxiety, and stress require careful evaluation without causal interpretation. This study examined their associations with continuous Online Shopping Addiction Scale (OSAS) scores after accounting for sociodemographic characteristics and online shopping frequency. A cross-sectional convenience sample included 438 adults in Türkiye (54.0% female among respondents reporting gender; mean age = 33.79, SD = 10.24); a total of 317 participated online and 121 completed printed questionnaires face to face. Pearson correlations were estimated in the full sample. Hierarchical regressions used a common complete-case sample (*n* = 375). Because residual diagnostics indicated heteroscedasticity and tail departures, HC3 robust standard errors and 95% confidence intervals were reported. Anxiety, depression, and stress showed statistically significant but modest correlations with OSAS total scores (r = 0.34, 0.32, and 0.27, respectively; all *p* < 0.001). In separate adjusted models, anxiety (ΔR^2^ = 0.070; β = 0.283), depression (ΔR^2^ = 0.068; β = 0.276), and stress (ΔR^2^ = 0.042; β = 0.217) each increased explained variance (all *p* < 0.001). In the combined model, the DASS-21 block added ΔR^2^ = 0.091; anxiety (β = 0.206, *p* = 0.002) and depression (β = 0.200, *p* = 0.001) had comparable independent associations, whereas stress did not (β = −0.050, *p* = 0.473). Online shopping frequency had the largest standardized coefficient among concurrent covariates (β = 0.280), but it is conceptually proximal to the OSAS outcome. The combined model explained 31.6% of the variance (adjusted R^2^ = 0.291). Recruitment-mode comparisons were small and nonsignificant after Holm correction. Depression and anxiety were independently associated with higher OSAS scores, while most outcome variance remained unexplained. The findings do not establish temporal prediction, causation, or clinical diagnosis and should be interpreted in light of self-report measurement, construct overlap, residual confounding, and the digitally engaged convenience sample.

## 1. Introduction

E-commerce has reduced temporal and geographic barriers to purchasing and created continuous exposure to shopping opportunities (Rose & Dhandayudham, 2014). Foundational reviews describe compulsive buying–shopping as persistent loss of control accompanied by distress or adverse consequences rather than high purchasing involvement alone (Black, 2007a, 2007b; Lejoyeux & Weinstein, 2010; Müller et al., 2015). Contemporary scholarship has debated whether hedonic consumption and technology-mediated addictive behaviors should be understood as related but distinguishable conditions and has emphasized interactions among person-level, affective, cognitive, and executive processes (Baggio et al., 2018; Brand et al., 2019; James et al., 2025). Problematic online shopping is commonly discussed as an online manifestation of compulsive buying–shopping disorder (CBSD), although whether it constitutes a distinct specific internet-use disorder remains debated (Mishra et al., 2023; Müller et al., 2021b). The OSAS operationalizes online-shopping-related symptoms through addiction components including salience/tolerance, mood modification, withdrawal, relapse, and conflict (Griffiths, 2005). CBSD is not a standalone diagnosis in DSM-5 and lacks dedicated ICD-11 diagnostic criteria; proposed expert-consensus criteria remain provisional and emphasize impaired control, persistence despite harm, distress, and functional impairment rather than purchase frequency alone (Müller et al., 2021a). Accordingly, the present study analyzes continuous OSAS scores and does not classify participants as clinically addicted.

Available instruments operationalize overlapping but nonidentical aspects of compulsive and problematic shopping, including the Bergen Shopping Addiction Scale, broader compulsive-buying measures, and the OSAS (Andreassen et al., 2015; Maraz et al., 2015; Ridgway et al., 2008; Zhao et al., 2017). Cross-language validation remains important when distinguishing online from offline manifestations (Chou et al., 2025), and factor-analytic evidence can be sensitive to nonnormality and model misspecification (Curran et al., 1996). Reported prevalence differs across instruments, settings, and populations, including general-population, shopping-center, student, young-adult, and Turkish samples (Cm et al., 2025; Koran et al., 2006; Maraz et al., 2016; Moon & Attiq, 2018; Ünübol et al., 2022; Villella et al., 2011). Compulsive buying may also vary with social context, as observed during the early COVID-19 period (Maraz & Yi, 2022). These differences reinforce the need to avoid deriving diagnostic or prevalence claims from a continuous scale score alone.

Clinical, community, and student research has associated compulsive or problematic buying with depression, anxiety, stress, impulsivity, self-esteem, self-control, financial attitudes, adverse childhood experiences, mood-regulation expectancies, social support, and coping (Aydin et al., 2021; David et al., 2024; Dixit et al., 2024; Erzincanlı et al., 2024; Kaur & Mearns, 2021; Lejoyeux et al., 1997; H. Li et al., 2022; Mishra et al., 2023; Otero-López & Villardefrancos, 2013, 2014; Zhang et al., 2016). Clinical comparisons further suggest overlap with other behavioral addictions, while treatment-seeking samples illustrate that substantial online shopping can occur within CBSD (Granero et al., 2016; Müller et al., 2019). Experimental studies indicate that depressed mood and mood-related impulsivity can influence buying-related responses (Kyrios et al., 2013; Nicolai et al., 2016), and anxiety sensitivity and anxiety symptoms have also been linked to compulsive buying (Gallagher et al., 2017; Weinstein et al., 2015). Stress-focused evidence includes cross-sectional associations, acute-stress cue-reactivity studies, and work on shifts from goal-directed behavior toward habits (Müller et al., 2025; Thomas et al., 2024, 2025; Trotzke et al., 2019; Zheng et al., 2020). Gender-related pathways and correlates are heterogeneous (Ching et al., 2016; Laskowski et al., 2024, 2025), and age-related findings in problematic internet use caution against attributing digital behavior to age alone (Ioannidis et al., 2018). Cross-sectional associations cannot determine temporal direction, and shared variance among depression, anxiety, and stress makes it important to distinguish separate from conditional associations.

Digital commerce introduces additional social and platform contexts. Influencer content, fear of missing out, materialism, social-media exposure, and video-platform affordances have been associated with conspicuous, impulsive, or compulsive consumption (Dinh & Lee, 2024; Jameel et al., 2024; Ngo et al., 2024; Pellegrino et al., 2022). Live-commerce traffic and induced-consumption practices may further intensify purchasing cues (C. Li et al., 2024), and online CBSD shows conceptual similarities to social-network-use disorder (Wegmann et al., 2023). Neuroimaging and decision-tree studies have explored individual-level prediction of OSAS-related outcomes (Shi et al., 2024; Wan et al., 2024), but predictive classification does not by itself establish causation, clinical diagnosis, or a platform effect.

Diagnostic and treatment evidence remains developing. Expert-consensus criteria and subsequent diagnostic evaluation emphasize impaired control, persistence, distress, and functional impairment (Müller et al., 2021a; Ni et al., 2026). A systematic review found that treatment research for CBSD remains limited (Müller et al., 2023), while intervention trials in the broader internet-use-disorder literature provide a related but not disorder-specific evidence base (Schmidt et al., 2022). Most directly for the present research question, Erzincanlı et al. (2024) simultaneously modeled DASS-21 depression, anxiety, and stress in a Turkish sample and found significant associations for depression and anxiety but not stress. The present study is therefore an independent replication and methodological extension: it provides hierarchical estimates adjusted for sociodemographic variables and online shopping frequency, compares separate with simultaneous DASS-21 models, describes correlations across the five OSAS subdimensions, and evaluates recruitment mode and the influence of including shopping frequency.

We hypothesized that depression, anxiety, and stress would each be positively associated with OSAS total and subscale scores. We then asked which distress dimensions would retain independent associations with OSAS total scores when entered simultaneously after the measured sociodemographic covariates and online shopping frequency. Because prior evidence suggested substantial overlap among the DASS-21 dimensions, no claim was made that one dimension would necessarily be meaningfully stronger than another.

## 2. Methods

### 2.1. Study Design

This cross-sectional, descriptive-correlational study examined associations between continuous OSAS scores and depression, anxiety, and stress. Data were collected through an anonymous Google Forms questionnaire and face-to-face administration of printed questionnaires. Eligible participants were adults aged 18 years or older who could read Turkish and voluntarily provided informed consent. Individuals who did not consent or submitted substantially incomplete forms were not included.

### 2.2. Ethical Considerations

The study was conducted in accordance with the principles of the Declaration of Helsinki. Ethical approval was obtained from Dokuz Eylül University Non-Interventional Studies Ethics Committee with the decision number 2025/23-01 on 9 July 2025. All participants provided informed consent before participation. Participation was voluntary, and participants were informed that they could withdraw from the study at any time. Data were collected anonymously and used only for scientific research purposes. Permissions for the use of the Depression Anxiety and Stress Scale-21 and the Online Shopping Addiction Scale were obtained from the relevant researchers.

### 2.3. Participants, Sampling Methodology, and Recruitment

Sample-size adequacy was evaluated in two ways. A G*Power 3.1 calculation for a two-tailed bivariate regression slope, with a standardized association of 0.15, α = 0.05, and power = 0.80, yielded a minimum target of 343 participants. A complementary design-sensitivity calculation aligned with the combined hierarchical regression used the fixed-model R^2^-increase framework. Assuming f^2^ = 0.15, α = 0.05, power = 0.80, three tested DASS-21 variables, and 13 total predictors, the minimum sample was 78. With the complete-case analytic sample of 375, the corresponding power exceeded 0.999. This sensitivity calculation evaluates adequacy under a prespecified medium incremental effect and is not an observed-effect post hoc power estimate.

A non-probability convenience strategy was used. The Google Forms questionnaire was distributed through Instagram, Twitter/X, LinkedIn, e-mail groups, and online announcements. Printed questionnaires were administered face to face to adults reached directly by the research team. Of 438 participants, 317 (72.4%) completed the electronic questionnaire and 121 (27.6%) completed a printed questionnaire. Recruitment mode was examined in score comparisons and as an additional covariate in a sensitivity model; these analyses do not remove the selection limitations of non-probability recruitment.

Participation was voluntary. Online participants completed an electronic consent form before accessing the survey, and face-to-face participants received the same consent information before completing the printed form. No names, identity numbers, addresses, telephone numbers, or IP addresses were collected. The retained Google Forms export included a unique submission timestamp for each online entry but did not contain survey start times, IP addresses, attention-check items, or a platform variable documenting cookie-based duplicate prevention. Consequently, IP filtering, completion-time screening, and attention-check exclusions were not performed. A post hoc comparison of complete response patterns within the retained online export, excluding timestamps, found no identical online response pattern. Paper responses were entered into the electronic database by the researchers and reviewed for completeness and internal inconsistency before analysis.

### 2.4. Sociodemographic and Behavioral Variables

The sociodemographic form recorded gender, age in years, education, marital status, employment status, and monthly income. Three participants did not disclose gender. For regression, education was represented by high school or below (reference), associate/bachelor’s degree, and graduate degree; marital status was represented as single/divorced (reference) versus married; and employment as not employed (reference) versus employed. Monthly income was dummy-coded with 0–20,000 TL as the reference. Online shopping frequency was coded on its original ordered five-point scale (1 = never, 2 = a few times a year, 3 = monthly, 4 = weekly, 5 = more than weekly), so its coefficient represents a one-category increase. Additional descriptive items assessed product categories, social-media use, perceived shopping behavior and change, feelings during shopping, and perceived psychological effects. Multiple-selection items were summarized as separate binary responses.

### 2.5. Measurement Instruments for Psychological Distress and Online Shopping Addiction

Depression Anxiety and Stress Scale-21: Depression, anxiety, and stress levels were assessed using the short form of the Depression Anxiety and Stress Scale-21. The original scale was developed by Lovibond and Lovibond in 1995 (Lovibond & Lovibond, 1995), and its Turkish validity and reliability study was conducted by Ö. Yılmaz et al. (2017). The scale was developed to measure symptoms of depression, anxiety, and stress experienced during the previous week. It consists of 21 items and three subscales: depression, anxiety, and stress. Each subscale includes seven items. Each item is rated on a four-point Likert-type scale ranging from 0 to 3, according to the severity or frequency of the symptom. The total score for each subscale ranges from 0 to 21, with higher scores indicating higher levels of depression, anxiety, or stress. In the original Turkish validation study, internal consistency coefficients ranged from 0.76 to 0.82 across the subscales. In the present study, depression, anxiety, and stress were evaluated using their respective subscale total scores. In the present sample, internal consistency was high for all three subscales (Cronbach’s α = 0.87 for anxiety, 0.91 for depression, and 0.92 for stress) and for the full 21-item scale (α = 0.95).

Online Shopping Addiction Scale: Online shopping addiction was assessed using the Turkish version of the Online Shopping Addiction Scale. The original scale was developed and validated by Zhao et al. (2017) to measure online shopping addiction as a specific form of internet-related behavioral addiction. The original instrument was constructed on the basis of Griffiths’ six-component model of behavioral addiction, including salience, mood modification, tolerance, withdrawal, conflict, and relapse, and consists of 18 items. In the original validation study, exploratory factor analysis, confirmatory factor analysis, and concurrent validity analyses supported the reliability and validity of the scale. The Turkish adaptation and psychometric evaluation of the scale were conducted by T. Yılmaz et al. (2022). The scale was designed to measure addictive tendencies related to online shopping behavior. It consists of 18 items and has a five-factor structure. The five subdimensions assess distinct facets of online shopping addiction derived from Griffiths’ components model: salience/tolerance (preoccupation with online shopping and its growing importance in daily life, together with the need for progressively greater engagement to achieve the desired effect); mood modification (use of online shopping to regulate, relieve, or escape negative emotional states); withdrawal (unpleasant affective states, such as feeling down, restless, or empty, when unable to shop online); relapse (repeated unsuccessful attempts to reduce or stop online shopping and a return to previous patterns); and conflict (interpersonal and functional problems, for example, arguments with family or reduced productivity at work or in education, arising from online shopping). Items are rated on a five-point Likert-type response format. Total scores range from 18 to 90, with higher scores indicating higher levels of online shopping addiction. In the Turkish validation study, the scale demonstrated a total internal consistency coefficient of 0.92 and a test–retest reliability of 0.79, with the five subscale coefficients ranging from 0.40 to 0.88. In the present sample, Cronbach’s α was 0.96 for the OSAS total score and ranged from 0.85 to 0.94 across the five subdimensions (salience/tolerance = 0.93, mood modification = 0.92, withdrawal = 0.90, relapse = 0.94, conflict = 0.85), indicating good-to-excellent internal consistency. OSAS total and subscale scores were analyzed continuously. No validated diagnostic cutoff was applied; the scale was used to quantify symptom tendencies and severity, not to diagnose a disorder or estimate prevalence.

### 2.6. Data Analysis

Descriptive analyses used all available observations (n = 438). Gender was missing for 3 participants, employment for 6, and income for 55; all other regression variables were complete. No values were imputed. Regression analyses used listwise deletion across the outcome and all variables in the final combined specification, producing a common analytic sample of n = 375 for every step and model. The three participants who did not disclose gender were retained in descriptive analyses but excluded from gender-adjusted regressions. Influence was evaluated using Cook’s distance; the maximum in the combined model was 0.028, and no case approached the conventional value of 1.0. Means, standard deviations, frequencies, and valid percentages were calculated as appropriate. Education was recoded into high school or below, associate/bachelor’s degree, and graduate degree. Shopping frequency was retained as the original five-level ordered score for the primary regression and was interpreted as a concurrent behavioral covariate rather than a temporally antecedent exposure.

Bivariate associations among the continuous study variables, specifically the three DASS-21 subscale scores (anxiety, depression, and stress) and the five OSAS subscale scores (salience/tolerance, mood modification, withdrawal, relapse, and conflict), were examined using Pearson product-moment correlation coefficients. The resulting correlation matrix was visualized as a heatmap to facilitate interpretation of the interrelationships among the psychological distress and online shopping addiction dimensions. Statistical significance of each correlation coefficient was evaluated at the *p* < 0.05, *p* < 0.01, and *p* < 0.001 levels. For survey items that permitted multiple selections (types of products purchased online and feelings experienced during online shopping), multiple response frequency analyses were conducted. Response frequencies and percentages (calculated as a proportion of the total number of respondents rather than total responses) were reported for each response category. These descriptive summaries provided a comprehensive overview of the behavioral and affective profiles associated with participants’ online shopping engagement. Hierarchical linear regression was used to quantify incremental associations with the OSAS total score. The same complete-case sample was used at all steps: gender (Step 1), age (Step 2), education dummies (Step 3), marital status (Step 4), employment (Step 5), income dummies (Step 6), shopping frequency (Step 7), and the DASS-21 variable or variables (Step 8). Three separate models entered anxiety, depression, or stress at Step 8; a combined model entered all three simultaneously. The separate-model increments represent each dimension’s overall association, including variance shared with the other dimensions, whereas the combined model estimates conditional independent associations. Variable ordering is an analytic adjustment sequence and does not establish temporal precedence.

*OSAS_i_ = β*_0_* + β*_1_*Female_i_ + β*_2_*Age_i_ + β*_3_*ᵀEducation_i_ + β*_4_*Married_i_ + β*_5_*Employed_i_ + β*_6_*ᵀIncome_i_ + β*_7_*ShoppingFrequency_i_ + β*_8_*ᵀDASS_i_ + ε_i_*

*For nested step s, ΔR^2^_s_ = R^2^_s_ − R^2^_(s−*1*)_. In separate models, DASS_i_ contained one subscale; in the combined model, it contained anxiety, depression, and stress.*

Final-model tables report B, HC3 heteroscedasticity-robust SEs and 95% confidence intervals for B, standardized β coefficients, exact *p* values, semi-partial correlations (sr), VIFs, and tolerances. Residual-versus-fitted and Q-Q plots, multivariable Breusch–Pagan tests, full rank-adjusted White tests, and Cook’s distances were examined. Breusch–Pagan tests were significant and Q-Q plots showed tail deviations, whereas the higher-dimensional White tests were not significant; coefficient inference therefore used HC3 SEs and confidence intervals. Recruitment-mode comparisons used Welch tests, Hedges’ *g*, and Holm-adjusted *p* values. Sensitivity analyses included recruitment mode as an additional covariate in the combined model and evaluated a combined model excluding shopping frequency to assess construct overlap. All statistical analyses were performed using IBM SPSS Statistics version 30.0 (IBM Corp., Armonk, NY, USA), STATA version 18.0 (StataCorp LLC, College Station, TX, USA), Python Version 3.10 (Python Software Foundation, Wilmington, DE, USA) with NumPy for matrix computations, and R version 4.5.3 (R Foundation for Statistical Computing, Vienna, Austria) using base R and the haven package. All statistical inference procedures were conducted using a two-tailed framework, and statistical significance was determined according to an a priori alpha threshold of 0.05, with *p*-values below 0.05 considered indicative of statistically significant evidence.

## 3. Results

A total of 438 participants were included (mean age = 33.79 years, SD = 10.24). Among the 435 participants reporting gender, 54.0% were female and 46.0% were male. Education was high school or below for 11.4%, associate/bachelor’s degree for 63.7%, and graduate degree for 24.9%; thus, 88.6% had associate-level or higher education. Active social-media use was reported by 91.8%. Online shopping occurred monthly for 34.5%, weekly for 23.7%, a few times per year for 21.0%, more than weekly for 19.6%, and never for 1.1%. Additional sample characteristics are shown in Table 1.

Online shopping preferences, social media platform use, and emotional responses of the participants are presented in Table 2. The most frequently purchased product category was clothing (71.7%), followed by food and beverages (45.2%), health and beauty products (39.7%), electronic products (34.0%), books/media (32.2%), and home decoration products (22.8%). Other product categories were reported by 6.2% of participants. Regarding feelings experienced during online shopping, 45.7% of participants reported feeling happy, 26.7% excited, and 11.9% normal or neutral. In addition, 7.8% reported feeling anxious or restless, 5.0% reported feeling lonely, and 3.0% reported other emotional responses.

Recruitment-mode differences were small and nonsignificant. Online versus face-to-face means (SDs) were 34.18 (16.12) versus 31.94 (16.13) for OSAS total, 4.01 (4.33) versus 3.50 (3.77) for anxiety, 4.40 (4.73) versus 4.83 (5.29) for depression, and 5.56 (5.20) versus 5.36 (5.02) for stress. The online-minus-face-to-face mean differences were 2.24 (95% CI [−1.16, 5.64]; Hedges’ *g* = 0.139), 0.51 ([−0.32, 1.33]; *g* = 0.121), −0.44 ([−1.52, 0.65]; *g* = −0.089), and 0.20 ([−0.87, 1.27]; *g* = 0.039), respectively. Holm-adjusted *p* values ranged from 0.782 to 0.854.

**Figure 1:**
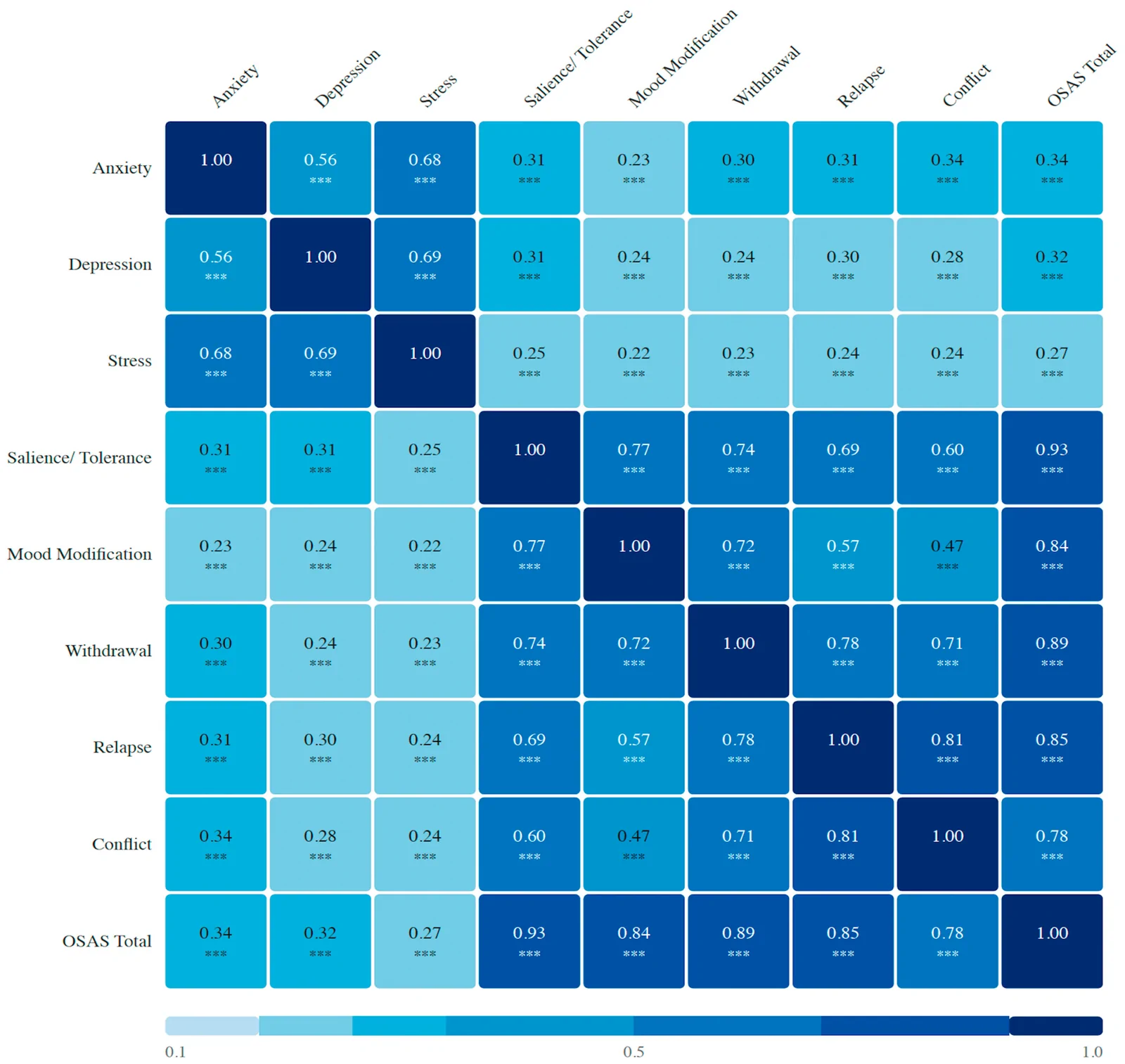
Correlation Matrix Showing the Relationships Between Anxiety, Depression, Stress, and Online Shopping Addiction Scale Total and Subscale Scores (*** *p* < 0.001).

The correlation matrix of anxiety, depression, stress, and online shopping addiction-related scores is presented in Figure 1. Anxiety was positively correlated with depression (r = 0.56, *p* < 0.001) and stress (r = 0.68, *p* < 0.001), while depression was positively correlated with stress (r = 0.69, *p* < 0.001). Anxiety, depression, and stress were also positively correlated with the OSAS total score, with correlation coefficients of 0.34, 0.32, and 0.27, respectively (all *p* < 0.001). With regard to the OSAS subdimensions, anxiety showed positive correlations with salience/tolerance (r = 0.31), mood modification (r = 0.23), withdrawal (r = 0.30), relapse (r = 0.31), and conflict (r = 0.34) (all *p* < 0.001). Depression was also positively correlated with salience/tolerance (r = 0.31), mood modification (r = 0.24), withdrawal (r = 0.24), relapse (r = 0.30), and conflict (r = 0.28) (all *p* < 0.001). Similarly, stress was positively correlated with salience/tolerance (r = 0.25), mood modification (r = 0.22), withdrawal (r = 0.23), relapse (r = 0.24), and conflict (r = 0.24) (all *p* < 0.001). Overall, all OSAS subdimensions demonstrated statistically significant positive correlations with anxiety, depression, and stress scores.

All hierarchical models used the common complete-case sample (n = 375). Residual Q-Q plots showed upper-tail departures (Q-Q correlations = 0.975–0.979), and residual skewness and excess kurtosis ranged from 0.84 to 0.92 and from 1.14 to 1.55, respectively. Multivariable Breusch–Pagan tests were significant in every final model (all *p* ≤ 0.006), whereas the full rank-adjusted White tests were not (*p* values = 0.057–0.345). Table 3, Table 4, Table 5 and Table 6 therefore report HC3 robust SEs, confidence intervals, and coefficient tests. Cook’s distances were small (maximum = 0.035 across the separate models and 0.028 in the combined model). In the anxiety model (Table 3), Step 8 increased R^2^ by 0.070 (ΔF(1, 363) = 36.02, *p* < 0.001), with final R^2^ = 0.295 and adjusted R^2^ = 0.274. Anxiety was positively associated with OSAS scores (B = 1.075, HC3 SE = 0.197, 95% CI [0.688, 1.462], β = 0.283, *p* < 0.001). Female gender (β = 0.113, *p* = 0.019), age (β = −0.160, *p* = 0.002), and shopping frequency (β = 0.300, *p* < 0.001) were also significant; education, marital status, employment, and income were not.

In the depression model (Table 4), Step 8 increased R^2^ by 0.068 (ΔF(1, 363) = 34.99, *p* < 0.001), with final R^2^ = 0.293 and adjusted R^2^ = 0.272. Depression was positively associated with OSAS scores (B = 0.896, HC3 SE = 0.172, 95% CI [0.558, 1.234], β = 0.276, *p* < 0.001). Female gender (β = 0.171, *p* < 0.001), age (β = −0.154, *p* = 0.005), and shopping frequency (β = 0.286, *p* < 0.001) were significant; education, marital status, employment, and income were not.

In the stress model (Table 5), Step 8 increased R^2^ by 0.042 (ΔF(1, 363) = 21.02, *p* < 0.001), with final R^2^ = 0.267 and adjusted R^2^ = 0.245. Stress was positively associated with OSAS scores in this separate model (B = 0.678, HC3 SE = 0.166, 95% CI [0.352, 1.004], β = 0.217, *p* < 0.001). Female gender (β = 0.124, *p* = 0.011), age (β = −0.163, *p* = 0.005), and shopping frequency (β = 0.314, *p* < 0.001) were significant; education, marital status, employment, and income were not.

In the combined model (Table 6), the three-variable DASS-21 block added ΔR^2^ = 0.091, ΔF(3, 361) = 15.95, *p* < 0.001; final R^2^ = 0.316; and adjusted R^2^ = 0.291. Anxiety (B = 0.783, HC3 SE = 0.246, 95% CI [0.299, 1.267], β = 0.206, sr = 0.145, *p* = 0.002) and depression (B = 0.650, HC3 SE = 0.198, 95% CI [0.260, 1.040], β = 0.200, sr = 0.136, *p* = 0.001) had comparable independent associations. Stress was not significant (B = −0.154, HC3 SE = 0.215, 95% CI [−0.578, 0.269], β = −0.050, sr = −0.031, *p* = 0.473). Female gender (β = 0.148, *p* = 0.002), younger age (β = −0.146, *p* = 0.004), and shopping frequency (β = 0.280, *p* < 0.001) were also associated with OSAS scores; the income block was nonsignificant, ΔR^2^ = 0.005, *p* = 0.532. Thus, 68.4% of unadjusted outcome variance (70.9% using adjusted R^2^) remained unaccounted for. DASS-21 VIFs were 2.00–2.60, and all model VIFs were 1.19–4.99 (tolerances = 0.20–0.84). Adding face-to-face mode to the combined model yielded a small nonsignificant coefficient (B = 1.361, HC3 SE = 1.812, β = 0.038, *p* = 0.453), while the DASS-21 estimates were essentially unchanged. When shopping frequency was omitted, anxiety (β = 0.225, *p* = 0.001) and depression (β = 0.249, *p* < 0.001) remained significant, whereas stress did not (β = −0.075, *p* = 0.287).

## 4. Discussion

In this cross-sectional sample, anxiety, depression, and stress each had modest positive correlations with OSAS total and subscale scores. Their separate adjusted increments were similar for anxiety (ΔR^2^ = 0.070) and depression (0.068) and smaller for stress (0.042); these increments include variance shared with the other distress dimensions and are not unique effects. In the combined model, anxiety and depression had comparable independent associations (β = 0.206 and 0.200), whereas stress did not. The model explained 31.6% of OSAS variance, leaving most variation unaccounted for. These results support an independent replication and extension of the pattern reported by Erzincanlı et al. (2024), but they do not establish temporal prediction or causation.

Women had higher adjusted OSAS scores in the combined model, but this sample-specific coefficient should not be interpreted as an inherent female vulnerability. Gender findings in the CBSD literature are heterogeneous (Laskowski et al., 2024), and contextual factors may contribute. Clothing was the most frequently purchased category in this sample (71.7%); gendered marketing, differential exposure to shopping cues, and personalized recommendation systems are plausible environmental explanations, but none was measured and product-by-gender mechanisms were not tested. Similarly, the negative age coefficient does not demonstrate that digital fluency explains the association because digital literacy, internet-use intensity, and technology engagement were not measured. Gender and age therefore describe adjusted associations in this sample, not diagnostic or stigmatizing risk-group labels.

Depression showed a positive adjusted association with OSAS scores in both separate and combined models, consistent with prior research on compulsive buying and depressed mood (Kyrios et al., 2013; Mishra et al., 2023). Although some argue that online shopping addiction reflects platform-induced consumption more than depression (C. Li et al., 2024), mood repair and self-medication remain plausible theoretical accounts; however, neither purchasing motives nor within-person mood change was measured here. The separate depression increment (ΔR^2^ = 0.068) should therefore be understood as its overall contribution within that model, including shared distress variance, rather than a unique causal pathway.

Anxiety likewise had a positive association with OSAS scores and a separate-model increment (ΔR^2^ = 0.070) that was practically similar to depression’s increment. Prior work has linked anxiety and anxiety sensitivity to compulsive buying (Gallagher et al., 2017; Weinstein et al., 2015). Negative reinforcement is a plausible theoretical hypothesis whereby purchasing temporarily reduces arousal, but that process was not measured or tested in the present study. Accordingly, the cross-sectional association should not be interpreted as evidence that anxiety causes problematic shopping.

Stress was significant when entered without the other DASS-21 dimensions but became nonsignificant in the combined model. The contrast between the separate-model coefficient (β = 0.217) and the conditional coefficient (β = −0.050) is consistent with substantial shared variance among depression, anxiety, and stress (intercorrelations r = 0.56–0.69). Semi-partial correlations in Table 6 quantify the conditional contributions: sr = 0.145 for anxiety, 0.136 for depression, and −0.031 for stress. This pattern does not show that stress acts through depression or anxiety; mediation or temporal mechanisms were not tested.

The results do not support ranking depression as meaningfully more important than anxiety. Their separate ΔR^2^ values differed by only 0.002, and their combined standardized coefficients were nearly identical. Both associations were modest, and the model left 68.4% of OSAS variance unexplained. Unmeasured factors such as impulsivity, materialism, self-control, financial strain, coping, and platform exposure may account for additional variance or confound the adjusted coefficients.

Online shopping frequency had the largest standardized coefficient among the concurrently measured covariates, but it is conceptually proximal to the OSAS outcome. Frequency may partly index behavioral intensity or overlap with engagement and escalation content rather than function as an independent etiological factor. The sensitivity model excluding frequency preserved the independent anxiety and depression associations, but it does not eliminate common-method bias. Neither cross-sectional frequency nor retrospectively reported increases in shopping can demonstrate progression or tolerance. Likewise, positive emotions during shopping and social-media exposure are descriptive findings; reinforcement processes and algorithmic effects were not tested. Because no interaction model was estimated, the findings do not support a synergistic interpretation.

### 4.1. Clinical, Social, and Platform-Level Implications

The findings concern continuous symptom scores and do not justify diagnosing or targeting demographic groups. Clinically, screening for distress may be considered only when a person reports impaired control, distress, or functional consequences, and it should be paired with assessment of financial and contextual factors. Broader implications include consumer protection, financial well-being, and responsible platform design. Behavioral targeting could create an ethical risk if signals correlated with psychological vulnerability are used to intensify purchasing prompts; however, algorithmic exposure and induced purchasing were not measured here. Candidate autonomy-preserving safeguards include user-configurable recommendation pauses or quiet hours, the option to disable one-click purchasing, cooling-off intervals, transparent personalization controls, and voluntary spending limits or alerts. None of these interventions was evaluated in this study. Their effectiveness, accessibility, privacy implications, equity, and unintended effects require co-designed experimental and implementation research.

### 4.2. Limitations

Several limitations constrain interpretation. First, the cross-sectional design cannot establish temporal precedence, mediation, or causality; reverse and bidirectional associations remain plausible. Second, all variables were self-reported concurrently. The OSAS was analyzed continuously without a validated clinical cutoff, so high scores cannot by themselves distinguish clinically significant CBSD from frequent or high-involvement but non-impairing shopping. Neither demographic membership nor shopping frequency constitutes a diagnosis. Third, convenience sampling overrepresented digitally engaged and highly educated adults: 72.4% participated online, 91.8% used social media actively, and 88.6% had associate-level or higher education. Nonsignificant mode comparisons do not remove selection bias, and the direction or magnitude of that bias cannot be inferred.

Fourth, the online export did not permit IP-based filtering, start-to-finish timing, or attention-check screening. Fifth, shopping frequency and OSAS were conceptually proximal and measured with the same method, creating potential criterion contamination; the omit-frequency sensitivity analysis does not fully resolve this issue. Sixth, important confounders were not measured, including impulsivity, materialism, self-control, neuroticism, coping strategies, psychiatric diagnoses, financial strain, and exposure to platform design. Residual confounding may therefore be substantial. Seventh, the combined model left most OSAS variance unexplained, and residual diagnostics required HC3 robust inference. Finally, the Turkish convenience sample limits cultural and population generalizability. Preregistered longitudinal, experimental, and probability-sampling studies using objective purchasing indicators and validated clinical assessment are needed.

## 5. Conclusions

In this cross-sectional study, depression and anxiety had comparable independent associations with higher continuous OSAS scores after adjustment for measured sociodemographic variables and shopping frequency; stress did not retain an independent association when the three distress dimensions were entered together. The correlations were modest, and the combined model left most outcome variance unexplained. Shopping frequency was concurrently associated with OSAS scores but overlapped conceptually with the outcome and should not be interpreted as a causal driver. Gender, age, and income findings are sample-specific associations, not diagnostic characteristics or grounds for labeling demographic groups. Because the OSAS was not used with a validated clinical cutoff, the study does not estimate disorder prevalence or distinguish diagnosis from high-involvement shopping. Future work should establish temporal direction, measure major confounders, use more representative samples and objective purchasing data, and prospectively test clinical, consumer-protection, and user-controlled platform interventions.

## Figures and Tables

**Table 1:** Sociodemographic Characteristics of the Research Group (n = 438).

Variables	*n* (%)/Mean ± SD
Gender (n = 435) *	
Female	235 (54.0)
Male	200 (46.0)
Age (years)	33.79 ± 10.24
Education Level	
High school or below	50 (11.4)
Associate or Bachelor’s degree	279 (63.7)
Graduate degree	109 (24.9)
Marital Status	
Single or divorced	248 (56.6)
Married	190 (43.4)
Employment Status (n = 432)	
Employed	324 (75.0)
Unemployed	108 (25.0)
Monthly Income (n = 383)	
0–20,000 TL	61 (15.9)
20,001–40,000 TL	81 (21.1)
40,001–60,000 TL	90 (23.5)
60,001 TL and above	151 (39.4)
Online Shopping Frequency	
Never	5 (1.1)
A few times a year	92 (21.0)
Once a month	151 (34.5)
Once a week	104 (23.7)
More than once a week	86 (19.6)
Active Social Media Use	
Yes	402 (91.8)
No	36 (8.2)
General View on Online Shopping	
Never shop	9 (2.1)
Shop rarely	135 (30.8)
Shop moderately	193 (44.1)
Shop frequently	86 (19.6)
Shop excessively	15 (3.4)
Change in Online Shopping Habits Over Time	
Decreased over time	71 (16.2)
No change	155 (35.4)
Increased over time	212 (48.4)
Perceived Psychological Impact of Online Shopping	
Negative effects	25 (5.7)
No effect	184 (42.0)
Positive effects	188 (42.9)
Do not know	41 (9.4)

* Three participants checked the box indicating they don’t want to specify their gender. Categorical variables are presented as n (%); continuous variables are presented as Mean ± SD. Percentages for categorical variables with missing data are calculated based on valid responses.

**Table 2:** Online Shopping Preferences, Social Media Platform Use, and Emotional Responses of the Participants.

Variables	*n*	*%*
Products purchased online ^a^ (N = 438)		
Clothing	314	71.7
Food and beverages	198	45.2
Health and beauty products	174	39.7
Electronic products	149	34.0
Books/media	141	32.2
Home decoration	100	22.8
Other	27	6.2
Feelings during online shopping ^b^ (N = 438)		
Happy	200	45.7
Excited	117	26.7
Normal/Neutral	52	11.9
Anxious/Restless	34	7.8
Lonely	22	5.0
Other	13	3.0

^a^ Multiple response item; percentages may exceed 100% as participants could select more than one option. ^b^ Open-ended responses were categorized into thematic groups. Coded response options included: Happy, Anxious/Restless, Excited, Lonely, and Normal. Free-text responses were recategorized into the closest thematic group or classified as Other.

**Table 3:** Final hierarchical regression model for OSAS total scores: DASS-21 anxiety entered at Step 8.

Variable		B	HC3 SE	95% CI for B	β	sr	*p*	VIF (Tol.)
Constant		16.214	4.536	[7.294, 25.135]	-	-	<0.001	-
Gender (female)		3.651	1.545	[0.613, 6.689]	0.113	0.107	0.019	1.12 (0.89)
Age (years)		−0.250	0.081	[−0.410, −0.090]	−0.160	−0.124	0.002	1.65 (0.61)
Education (associate/bachelor’s)		1.156	2.258	[−3.285, 5.597]	0.035	0.021	0.609	2.75 (0.36)
Education (graduate)		−0.262	2.510	[−5.199, 4.674]	−0.007	−0.004	0.917	2.78 (0.36)
Marital status (married)		2.571	1.606	[−0.587, 5.730]	0.080	0.071	0.110	1.24 (0.80)
Employment (employed)		−0.774	2.334	[−5.364, 3.816]	−0.020	−0.014	0.740	1.93 (0.52)
Income (20,001–40,000 TL)		4.689	2.666	[−0.553, 9.932]	0.119	0.073	0.079	2.65 (0.38)
Income (40,001–60,000 TL)		4.478	3.015	[−1.452, 10.407]	0.119	0.062	0.138	3.64 (0.27)
Income (60,001 TL or more)		2.839	2.891	[−2.846, 8.524]	0.087	0.039	0.327	4.92 (0.20)
Online shopping frequency		4.527	0.748	[3.056, 5.998]	0.300	0.274	<0.001	1.20 (0.83)
DASS-21 anxiety		1.075	0.197	[0.688, 1.462]	0.283	0.265	<0.001	1.14 (0.88)
**Hierarchical model-fit statistics**								
**Step**	**Variable(s) added**	**R^2^**	**Adjusted R^2^**	**ΔR^2^**	**ΔF (df^1^, df^2^)**		***p***	**Predictors**
1	Gender	0.046	0.043	0.046	17.78 (1, 373)		<0.001	1
2	+Age	0.108	0.103	0.062	25.91 (1, 372)		<0.001	2
3	+Education	0.111	0.101	0.003	0.71 (2, 370)		0.492	4
4	+Marital status	0.126	0.114	0.015	6.14 (1, 369)		0.014	5
5	+Employment	0.127	0.113	0.001	0.55 (1, 368)		0.461	6
6	+Income	0.132	0.111	0.005	0.73 (3, 365)		0.532	9
7	+Shopping frequency	0.225	0.204	0.093	43.61 (1, 364)		<0.001	10
8	+DASS-21 variable(s)	0.295	0.274	0.070	36.02 (1, 363)		<0.001	11

Analytic n = 375 complete cases. The coefficient section presents the final step. B = unstandardized coefficient; HC3 SE = heteroscedasticity-robust standard error; CI = confidence interval for B; β = standardized coefficient; sr = semi-partial correlation; VIF = variance inflation factor; Tol. = tolerance. Reference categories: male; high school or below; single/divorced; not employed; income 0–20,000 TL. Shopping frequency was coded 1–5. ΔR^2^ is the increase from the preceding nested step.

**Table 4:** Final hierarchical regression model for OSAS total scores: DASS-21 depression entered at Step 8.

Variable		B	HC3 SE	95% CI for B	β	sr	*p*	VIF (Tol.)
Constant		15.593	4.533	[6.679, 24.507]	-	-	<0.001	-
Gender (female)		5.506	1.503	[2.550, 8.462]	0.171	0.161	<0.001	1.13 (0.88)
Age (years)		−0.240	0.084	[−0.406, −0.075]	−0.154	−0.119	0.005	1.65 (0.61)
Education (associate/bachelor’s)		2.206	2.106	[−1.936, 6.348]	0.067	0.040	0.296	2.71 (0.37)
Education (graduate)		0.489	2.320	[−4.074, 5.052]	0.013	0.008	0.833	2.76 (0.36)
Marital status (married)		3.017	1.612	[−0.153, 6.187]	0.093	0.084	0.062	1.24 (0.80)
Employment (employed)		1.050	2.567	[−3.998, 6.098]	0.027	0.019	0.683	1.98 (0.51)
Income (20,001–40,000 TL)		2.125	2.758	[−3.299, 7.548]	0.054	0.033	0.442	2.67 (0.38)
Income (40,001–60,000 TL)		1.569	3.139	[−4.604, 7.742]	0.042	0.022	0.617	3.61 (0.28)
Income (60,001 TL or more)		−0.009	3.071	[−6.047, 6.030]	−0.000	−0.000	0.998	4.86 (0.21)
Online shopping frequency		4.327	0.729	[2.893, 5.761]	0.286	0.260	<0.001	1.21 (0.82)
DASS-21 depression		0.896	0.172	[0.558, 1.234]	0.276	0.261	<0.001	1.12 (0.89)
**Hierarchical model-fit statistics**								
**Step**	**Variable(s) added**	**R^2^**	**Adjusted R^2^**	**ΔR^2^**	**ΔF (df^1^, df^2^)**		***p***	**Predictors**
1	Gender	0.046	0.043	0.046	17.78 (1, 373)		<0.001	1
2	+Age	0.108	0.103	0.062	25.91 (1, 372)		<0.001	2
3	+Education	0.111	0.101	0.003	0.71 (2, 370)		0.492	4
4	+Marital status	0.126	0.114	0.015	6.14 (1, 369)		0.014	5
5	+Employment	0.127	0.113	0.001	0.55 (1, 368)		0.461	6
6	+Income	0.132	0.111	0.005	0.73 (3, 365)		0.532	9
7	+Shopping frequency	0.225	0.204	0.093	43.61 (1, 364)		<0.001	10
8	+DASS-21 variable(s)	0.293	0.272	0.068	34.99 (1, 363)		<0.001	11

Analytic n = 375 complete cases. The coefficient section presents the final step. B = unstandardized coefficient; HC3 SE = heteroscedasticity-robust standard error; CI = confidence interval for B; β = standardized coefficient; sr = semi-partial correlation; VIF = variance inflation factor; Tol. = tolerance. Reference categories: male; high school or below; single/divorced; not employed; income 0–20,000 TL. Shopping frequency was coded 1–5. ΔR^2^ is the increase from the preceding nested step.

**Table 5:** Final hierarchical regression model for OSAS total scores: DASS-21 stress entered at Step 8.

Variable		B	HC3 SE	95% CI for B	β	sr	*p*	VIF (Tol.)
Constant		16.546	4.777	[7.153, 25.940]	-	-	<0.001	-
Gender (female)		3.991	1.569	[0.906, 7.077]	0.124	0.117	0.011	1.12 (0.90)
Age (years)		−0.256	0.090	[−0.432, −0.080]	−0.163	−0.127	0.005	1.65 (0.61)
Education (associate/bachelor’s)		1.293	2.377	[−3.382, 5.967]	0.039	0.023	0.587	2.77 (0.36)
Education (graduate)		−0.448	2.631	[−5.622, 4.726]	−0.012	−0.007	0.865	2.81 (0.36)
Marital status (married)		2.397	1.625	[−0.798, 5.593]	0.074	0.066	0.141	1.25 (0.80)
Employment (employed)		0.254	2.542	[−4.745, 5.253]	0.007	0.005	0.920	1.96 (0.51)
Income (20,001–40,000 TL)		3.756	2.688	[−1.530, 9.041]	0.095	0.058	0.163	2.64 (0.38)
Income (40,001–60,000 TL)		2.848	3.101	[−3.251, 8.948]	0.075	0.040	0.359	3.60 (0.28)
Income (60,001 TL or more)		0.872	2.915	[−4.861, 6.605]	0.027	0.012	0.765	4.86 (0.21)
Online shopping frequency		4.748	0.764	[3.246, 6.249]	0.314	0.288	<0.001	1.19 (0.84)
DASS-21 stress		0.678	0.166	[0.352, 1.004]	0.217	0.206	<0.001	1.11 (0.90)
**Hierarchical model-fit statistics**								
**Step**	**Variable(s) added**	**R^2^**	**Adjusted R^2^**	**ΔR^2^**	**ΔF (df^1^, df^2^)**		***p***	**Predictors**
1	Gender	0.046	0.043	0.046	17.78 (1, 373)		<0.001	1
2	+Age	0.108	0.103	0.062	25.91 (1, 372)		<0.001	2
3	+Education	0.111	0.101	0.003	0.71 (2, 370)		0.492	4
4	+Marital status	0.126	0.114	0.015	6.14 (1, 369)		0.014	5
5	+Employment	0.127	0.113	0.001	0.55 (1, 368)		0.461	6
6	+Income	0.132	0.111	0.005	0.73 (3, 365)		0.532	9
7	+Shopping frequency	0.225	0.204	0.093	43.61 (1, 364)		<0.001	10
8	+DASS-21 variable(s)	0.267	0.245	0.042	21.02 (1, 363)		<0.001	11

Analytic n = 375 complete cases. The coefficient section presents the final step. B = unstandardized coefficient; HC3 SE = heteroscedasticity-robust standard error; CI = confidence interval for B; β = standardized coefficient; sr = semi-partial correlation; VIF = variance inflation factor; Tol. = tolerance. Reference categories: male; high school or below; single/divorced; not employed; income 0–20,000 TL. Shopping frequency was coded 1–5. ΔR^2^ is the increase from the preceding nested step.

**Table 6:** Final hierarchical regression model for OSAS total scores: anxiety, depression, and stress entered together at Step 8.

Variable		B	HC3 SE	95% CI for B	β	sr	*p*	VIF (Tol.)
Constant		14.754	4.451	[6.000, 23.508]	-	-	0.001	-
Gender (female)		4.765	1.525	[1.766, 7.764]	0.148	0.136	0.002	1.19 (0.84)
Age (years)		−0.229	0.080	[−0.386, −0.072]	−0.146	−0.114	0.004	1.66 (0.60)
Education (associate/bachelor’s)		1.448	2.124	[−2.729, 5.625]	0.044	0.026	0.496	2.79 (0.36)
Education (graduate)		−0.042	2.379	[−4.721, 4.637]	−0.001	−0.001	0.986	2.82 (0.35)
Marital status (married)		2.856	1.599	[−0.288, 6.000]	0.088	0.079	0.075	1.26 (0.80)
Employment (employed)		0.376	2.451	[−4.445, 5.196]	0.010	0.007	0.878	1.99 (0.50)
Income (20,001–40,000 TL)		3.273	2.686	[−2.010, 8.556]	0.083	0.050	0.224	2.72 (0.37)
Income (40,001–60,000 TL)		3.201	3.035	[−2.766, 9.169]	0.085	0.044	0.292	3.70 (0.27)
Income (60,001 TL or more)		1.729	2.947	[−4.067, 7.525]	0.053	0.024	0.558	4.99 (0.20)
Online shopping frequency		4.224	0.732	[2.785, 5.662]	0.280	0.253	<0.001	1.22 (0.82)
DASS-21 anxiety		0.783	0.246	[0.299, 1.267]	0.206	0.145	0.002	2.00 (0.50)
DASS-21 depression		0.650	0.198	[0.260, 1.040]	0.200	0.136	0.001	2.17 (0.46)
DASS-21 stress		−0.154	0.215	[−0.578, 0.269]	−0.050	−0.031	0.473	2.60 (0.38)
**Hierarchical model-fit statistics**								
**Step**	**Variable(s) added**	**R^2^**	**Adjusted R^2^**	**ΔR^2^**	**ΔF (df^1^, df^2^)**		***p***	**Predictors**
1	Gender	0.046	0.043	0.046	17.78 (1, 373)		<0.001	1
2	+Age	0.108	0.103	0.062	25.91 (1, 372)		<0.001	2
3	+Education	0.111	0.101	0.003	0.71 (2, 370)		0.492	4
4	+Marital status	0.126	0.114	0.015	6.14 (1, 369)		0.014	5
5	+Employment	0.127	0.113	0.001	0.55 (1, 368)		0.461	6
6	+Income	0.132	0.111	0.005	0.73 (3, 365)		0.532	9
7	+Shopping frequency	0.225	0.204	0.093	43.61 (1, 364)		<0.001	10
8	+DASS-21 variable(s)	0.316	0.291	0.091	15.95 (3, 361)		<0.001	13

Analytic n = 375 complete cases. The coefficient section presents the final step. B = unstandardized coefficient; HC3 SE = heteroscedasticity-robust standard error; CI = confidence interval for B; β = standardized coefficient; sr = semi-partial correlation; VIF = variance inflation factor; Tol. = tolerance. Reference categories: male; high school or below; single/divorced; not employed; income 0–20,000 TL. Shopping frequency was coded 1–5. ΔR^2^ is the increase from the preceding nested step.

## Data Availability

The datasets used and/or analyzed in this study are available upon reasonable request from the corresponding author (M.E.A.).
